# Digital Twin of the Female Pelvic Floor

**DOI:** 10.4236/ojog.2024.1411138

**Published:** 2024-11-06

**Authors:** Vladimir Egorov

**Affiliations:** Advanced Tactile Imaging, Inc., Ewing, NJ, USA

**Keywords:** Digital Twin, Female Pelvic Floor, Incontinence, Prolapse, Pelvic Pain, Endometriosis, Adenomyosis, Uterine Fibroids, Maternal Injury during Childbirth, Spontaneous Preterm Birth

## Abstract

Digital twin technology, originally developed for intricate physical systems, holds great potential in women’s healthcare, particularly in the management of pelvic floor disorders. This paper delves into the development of a digital twin specifically for the female pelvic floor, which can amalgamate various data sources such as imaging, biomechanical assessments, and patient-reported outcomes to offer personalized diagnostic and therapeutic insights. Through the utilization of 3D modeling and machine learning, the digital twin may facilitate precise visualization, prediction, and individualized treatment planning. Nevertheless, it is crucial to address the ethical and practical challenges related to data privacy and ensuring fair access. As this technology progresses, it has the potential to revolutionize gynecological and obstetric care by enhancing diagnostics, customizing treatments, and increasing patient involvement.

## 1. Introduction

The concept of a digital twin has evolved significantly since its initial applications in the aerospace industry, where it offered an innovative approach to monitoring and managing complex systems. Its value was underscored during NASA’s implementation of digital twins in the Apollo 13 mission, allowing engineers to simulate and resolve critical issues from Earth in real time [1]. Dr. Michael Grieves further formalized this concept in 2002, proposing a framework that integrates physical and virtual entities through a shared data interface [2]. This approach laid the foundation for the adaptability of digital twins across various sectors, including healthcare, where they are increasingly used to model and manage complex physiological systems.

In healthcare, digital twins have shown significant promise in managing chronic conditions, optimizing rehabilitation, and personalizing cardiovascular treatment [3]-[7]. For example, in diabetes management, digital twins offer a personalized approach, enabling continuous glucose monitoring and predictive analytics to prevent complications [4]. Similarly, digital twins allow for remote monitoring and personalized treatment adjustments in respiratory conditions like asthma, improving patient outcomes [5]. These examples highlight the versatility of digital twins, not only in managing chronic conditions but also in optimizing individual patient care.

Expanding on these advancements, a digital twin tailored for the female pelvic floor could revolutionize women’s health. Pelvic floor disorders, including incontinence, organ prolapse, chronic pain, and obstetric trauma, have significant impacts on women’s quality of life. Traditional diagnostic methods often fail to capture the complexity of these conditions, resulting in fragmented care. This manuscript aims to explore the potential of a digital twin to address these diagnostic and therapeutic challenges, emphasizing its role in integrating diverse data sources, improving diagnostic accuracy, and facilitating personalized treatment plans.

## 2. Digital Twin Framework for the Female Pelvic Floor

**Figure 1.**
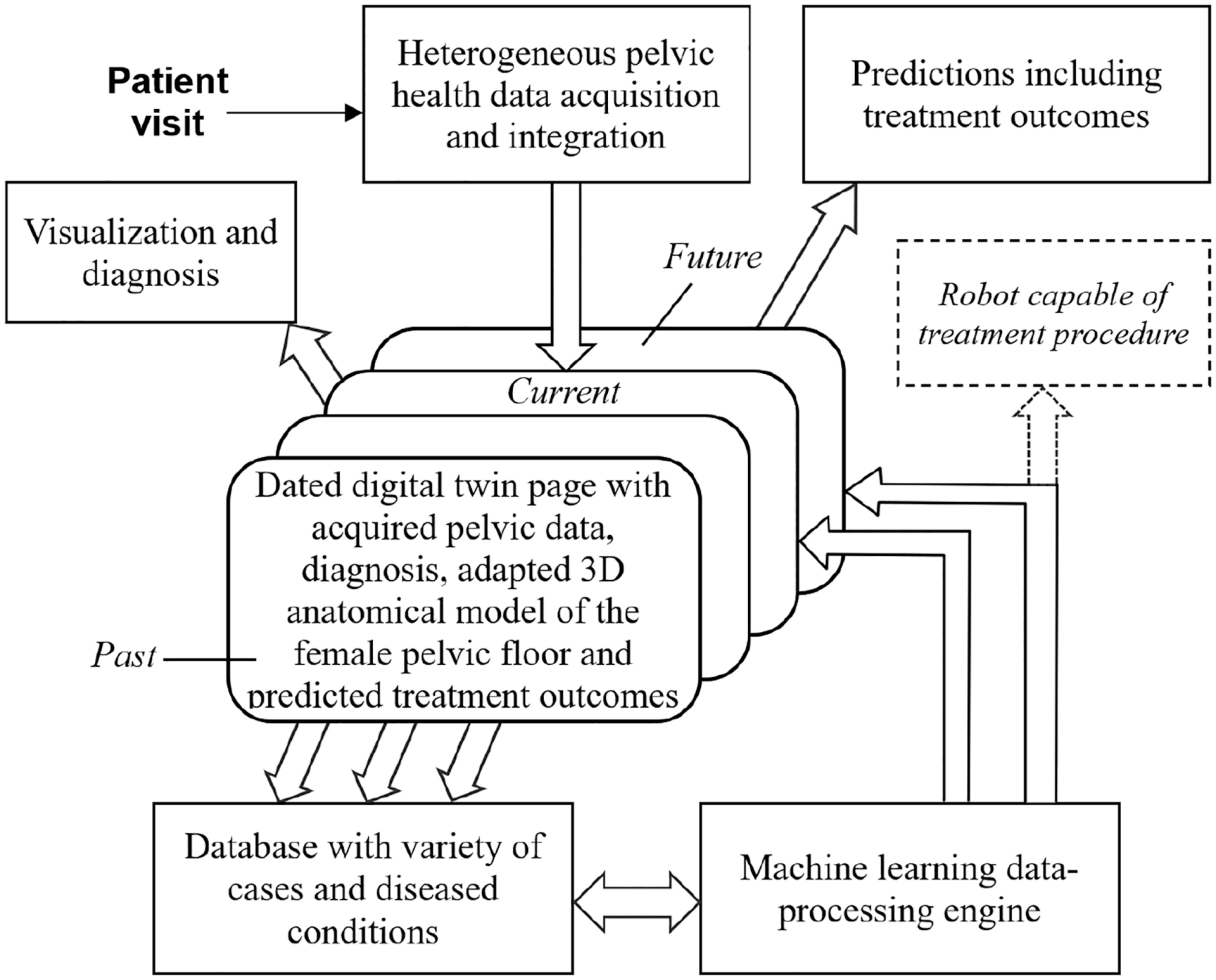
A schematic representation showcasing the fundamental elements of a digital twin for the female pelvic floor.

To realize a digital twin for the female pelvic floor, a robust technological framework must be established, incorporating comprehensive data collection, machine learning, and 3D anatomical modeling. This section outlines the key components of such a digital twin (see Figure 1).

### 2.1. Data Collection and Integration

The foundation of a reliable digital twin lies in the thorough and continuous collection of patient-specific data. For pelvic floor applications, this includes both objective and subjective data. Objective data sources encompass imaging modalities such as ultrasound, MRI, and CT scans, as well as physiological tests like urodynamic studies and biomechanical mapping of soft tissues [8]. These objective measurements provide insights into the structural and functional aspects of the pelvic floor, allowing for a detailed baseline representation within the digital twin.

Subjective data, on the other hand, consists of patient-reported outcomes, including symptom descriptions, quality-of-life assessments, and lifestyle factors. Collecting subjective data is essential for a holistic model, as pelvic floor disorders often involve symptoms and experiences unique to each patient. Integrating these two data types enables the digital twin to reflect a patient’s health status dynamically, adapt to changes over time, and support individualized treatment planning.

### 2.2. Machine Learning and Predictive Analytics

Machine learning algorithms [9] may be crucial in analyzing the vast data sets incorporated into the digital twin. By applying predictive analytics, these algorithms can identify patterns within a patient’s physiological and symptomatic data, offering insights into disease progression and treatment efficacy. For example, a machine learning model could predict how pelvic floor muscles respond to a specific therapeutic intervention, such as pelvic muscle exercises or surgical repair [10]. These predictive capabilities are valuable for clinicians and patients, providing a data-driven foundation for treatment decisions and long-term care planning.

Additionally, machine learning enhances the digital twin’s capacity for continuous learning. As new data are incorporated, the algorithms can refine their predictive models, improving accuracy. This iterative process is particularly beneficial in the context of chronic pelvic floor disorders, where ongoing monitoring and adaptive treatment strategies are essential.

### 2.3. Diagnostic Potential

The digital twin’s ability to integrate multiple data types into a cohesive model may offer unprecedented diagnostic potential for pelvic floor disorders. Traditional diagnostic tools, such as physical exams and imaging studies, provide isolated snapshots of a patient’s condition, which may not fully capture the complex interactions between muscles, connective tissues, and nerves within the pelvic floor. A digital twin may synthesize data from various diagnostic modalities, presenting a comprehensive view of the pelvic anatomy and function.

For instance, biomechanical mapping can quantify tissue elasticity and muscle strength, while urodynamic studies assess bladder and urethral function [11] [12]. Combining these data can allow the digital twin to generate a precise, patient-specific model that visualizes current dysfunctions and predicts future complications. This holistic diagnostic approach enables clinicians to identify early indicators of conditions like pelvic organ prolapse or incontinence, facilitating earlier interventions that may prevent further deterioration.

Another application is diagnosing complex pelvic floor conditions such as endometriosis, adenomyosis, and uterine fibroids [13]-[15]. Traditional diagnostic methods for these conditions often may not fully capture the extent of tissue abnormalities or disease progression. By synthesizing data from various imaging modalities with patient-reported symptoms and physiological metrics, the digital twin can construct a detailed 3D representation of the pelvic anatomy, enabling clinicians to detect subtle variations in tissue density, structural abnormalities, and lesion patterns associated with these conditions. For example, in endometriosis, the digital twin can help identify lesions and adhesions within the pelvic cavity that may otherwise be missed during routine examinations.

Over 85% of women experience obstetric trauma affecting one or more pelvic floor components, such as the perineal body, levator ani muscles, and the anal sphincter complex [16]. Damage to these structures is a significant, modifiable risk factor for subsequent pelvic floor disorders. Digital twin technology may help prevent or mitigate maternal injury during childbirth. Another clinical application of the digital twin may involve detecting conditions that lead to spontaneous preterm birth [17].

### 2.4. Therapeutic Applications

The digital twin not only may serve as a diagnostic tool but also can have significant implications for personalized therapeutic interventions. One of the main challenges in treating pelvic floor disorders is the variation in patient response to treatments. The digital twin can help address this issue by simulating different treatment scenarios. This will provide clinicians with a prediction of the potential outcomes of interventions before applying them. For example, the digital twin can be used to model the effects of pelvic floor physical therapy, surgical repair, or pharmaceutical treatments on a patient’s specific anatomy, predicting both immediate and long-term effects.

Additionally, the digital twin can support robotic-assisted treatments. By guiding robotic systems with predictive analytics, the digital twin may enable highly precise, patient-specific interventions. This capability is particularly valuable in surgical contexts, where anticipating tissue responses can reduce risks and improve recovery times. Furthermore, as the digital twin is updated with longitudinal data, it may enable real-time treatment monitoring, allowing clinicians to adjust therapeutic plans based on the patient’s evolving condition.

### 2.5. Patient Empowerment and Engagement

The digital twin of the female pelvic floor not only supports clinical decision-making but also serves as a powerful tool for enhancing patient engagement. It can provide a tangible, interactive representation of pelvic health, empowering patients to better understand their conditions and the impacts of various treatments. For instance, patients can visualize their pelvic floor anatomy in three dimensions, observing structural details and understanding how specific issues, such as muscle weakness or connective tissue dysfunction, affect their symptoms and overall well-being.

Engaging patients in this way fosters a more collaborative approach to healthcare. With the digital twin, clinicians could involve patients directly in the treatment planning process, showing them the potential outcomes of different interventions. This transparency promotes shared decision-making, as patients can see, for example, how pelvic floor exercises might enhance muscle support or how a surgical procedure could restore anatomical function. Research suggests that when patients are actively involved in their healthcare decisions, they are more likely to adhere to treatment protocols and achieve better health outcomes [18]. The digital twin thus aligns with broader trends in personalized and participatory medicine, where patients are encouraged to take an active role in managing their health.

Additionally, the digital twin can facilitate remote consultations and virtual follow-ups [19]. By integrating data from wearable devices, patients can continuously monitor relevant metrics, such as activity levels, muscle strength, or bladder function, through their digital twin. These data enable clinicians to track progress in real time and make timely adjustments to treatment plans, even from a distance [20]. This model may enhance the continuity of care and give patients the flexibility to engage with their healthcare outside traditional clinical settings.

### 2.6. Challenges and Ethical Considerations

While the digital twin offers significant potential for advancing women’s healthcare, its implementation raises several ethical and practical challenges. Data privacy is a primary concern, as the digital twin relies on continuously collecting and integrating sensitive patient information. Ensuring this data is securely stored, transmitted, and accessed is essential for maintaining patient trust. Moreover, given the intimate nature of pelvic floor health data, patients may be susceptible to how their information is handled. Adopting robust data protection measures and complying with healthcare privacy regulations, such as HIPAA in the United States or GDPR in the European Union, is crucial for safeguarding patient rights [21].

Another ethical consideration relates to the role of artificial intelligence and autonomy in medical decision-making. The digital twin’s machine learning algorithms analyze patient data to predict treatment outcomes and guide clinical interventions. However, reliance on these algorithms raises questions about the transparency of AI-driven recommendations and the potential for algorithmic bias. Ensuring that the digital twin’s algorithms are interpretable and explainable is necessary for clinicians and patients to trust its recommendations [22]. Furthermore, as autonomous robotic systems are introduced, establishing boundaries for human oversight becomes essential. Clinicians should have the ultimate authority in decision-making, with the digital twin serving as an assistive tool rather than a substitute for human expertise.

The integration of digital twins in healthcare also invites societal debate about the broader implications of these technologies. As digital twins become more prevalent, their influence on healthcare access and equity warrants consideration. For instance, if the costs associated with digital twin technology limit its availability to certain healthcare settings or patient populations, disparities in access to advanced diagnostics and treatments could arise [23]. Addressing these challenges requires a collaborative approach involving healthcare providers, policymakers, technologists, and patient advocacy groups. Together, they can establish guidelines that promote ethical use, equitable access, and societal acceptance of digital twin technology.

## 3. Conclusions

The concept of a digital twin for the female pelvic floor presents an innovative approach to diagnosing, treating, and managing complex pelvic health disorders. By integrating various data sources and utilizing advanced machine learning and 3D modeling techniques, the digital twin has the potential to provide valuable insights into the anatomical and functional aspects of the female pelvic floor. This integration enables personalized diagnostics and customized therapeutic interventions, addressing the challenges associated with conditions such as incontinence, prolapse, chronic pelvic pain, endometriosis, adenomyosis, and uterine fibroids. Furthermore, the digital twin technology may contribute to preventing maternal injury during childbirth and improving the early detection of conditions that can lead to spontaneous preterm birth.

Additionally, the digital twin may empower patients by offering a visual and interactive representation of pelvic health, fostering engagement and shared decision-making, and encouraging active participation in treatment. As the technology advances, its potential applications may expand to include preventative healthcare and remote monitoring support. For example, by monitoring changes in pelvic floor function over time, the digital twin could aid in identifying early indicators of potential disorders, allowing for timely intervention and better long-term outcomes.

The widespread adoption of digital twins in women’s healthcare presents several challenges. It is crucial to prioritize data privacy, address ethical considerations related to AI and robotic autonomy, and ensure equitable access. Collaborative efforts among stakeholders are essential to integrate this technology into clinical practice responsibly. With these challenges in mind, the digital twin has the potential to redefine the standard of care for women’s pelvic health, ushering in a new era of personalized, data-driven healthcare. This technology holds the promise of improving clinical outcomes and aligns with the broader shift toward precision medicine, where treatment is customized to the individual’s unique characteristics and needs.
